# Experiments on Mesmerism

**Published:** 1842-07

**Authors:** 


### THE WESTERN JOURNAL.

Vol. VI.—No. I.

LOUISVILLE, JULY 1, 1842.

### TWO EVENINGS' EXPERIMENTS ON MESMERISM.

*By the Senior Editor.*

Having been informed that several gentleman in Cincinnati were practising Mesmerism, and had discovered a number of individuals who were susceptible to its influence, I determined to witness, for the first time, some of its alleged phenomena. Of these gentlemen, Mr. Elijah Burdsal, druggist, was said to be the most successful mesmeriser, and Mr. George Selves, innkeeper, one of those most easily thrown into a state of somnambulism, or what might more appropriately be called somniloquism. On applying to these gentlemen, they obligingly complied with my request, and the time appointed was Saturday evening, May 7th, 1842. Desirous of having the experiments conducted in a manner best calculated to bring out decisive results, I invited William Greene, Esq., and Mr. William R. Foster, who had made themselves familiar with mesmeric processes, and were firm believers in the reality of the effects said to be produced, and that nothing should be left entirely to my own observation and decision, or reported on my own veracity, I requested Messrs. James Hall, Samuel E. Foote and Robert Buchanan, to attend as observers and recorders of the phenomena—these gentlemen having assured me that they had made up no opinion on the subject of mesmerism. Messrs. E. D. Mansfield, Peyton S. Symmes, Alexander H. McGuffey and Dr. John A. Warder, and several ladies, were present as spectators.

On the arrival of the different gentlemen, at the house of Mr. McGuffey, I stated that my object would not be to ascertain the reality of the mesmeric sleep, *which I should grant*, but, whether Mr. Selves, while in that condition, could feel impressions made on my organs of sense and general feeling, when I should be placed in connection with him.

On examining the pulse of Mr. S., immediately after walking, it beat 96 in a minute; his health was good; his age between 30 and 40; his temperament lymphatic.

Mr. Burdsal commenced his manipulations 10 minutes after 8 o'-clock; and in five minutes pronounced Mr. S. asleep. My left hand was then placed in his, and after a few manipulations I was directed to put a question to Mr. S., which he answered, when Mr. B. pronounced the connection perfect. For the next hour and a half our hands remained in unbroken contact, and I stood by his side. Before any experiments were commenced, and when Mr. S. was not touched, it was observable, that his muscular system was unquiet, and that he moved his limbs, more or less, and also the muscles of his face, giving to his features more or less of distortion.

*Experiments on Muscular Motion and Muscular Sensation.*

1. Standing so far behind the subject, that if his eyes had not been closed, I could not have been distinctly seen, I took up one of the gentlemen with my right arm, and held him as long as it was possible; but Mr. S. gave no indication of sympathy with me in the effort. He did not exhibit more muscular uneasiness than before the experiments, and when interrogated did not complain.

2. One of the gentlemen then placed himself on my shoulder, and at length Mr. S. seemed to shrink away, and raising his left hand, appeared to point towards his shoulder. On being questioned he answered, inarticulately, but in tones which indicated oppression or uneasiness.

*Experiments on Respiration.*

1. Standing by his side, I breathed with great rapidity for about a minute, during which he raised his left hand toward his head, and also extended his arm; but his breathing remained tranquil.

2. In the same position, I suspended my breath for a minute, at the end of which time I felt deep anxiety in the chest, but he remained quite tranquil.

*Experiments on the sense of feeling in reference to pain.*

1. My right arm was rubbed with sand paper, when he gave evidence, by his restlessness and murmurs, of feeling pain; the sand paper, which made a noise, was then rubbed on the coat sleeve of a gentleman by my side, and my own arm was rubbed with a piece of fur, which appeared to affect him in the same manner as the sand paper; indeed his contortions and writhings at length became very great, and continued as long as the friction on the coat was kept up.

2. Dr. Warder then made a great number of punctures with a lancet on my arm, so deep as to bleed, during which the subject displayed some contortions of the mouth, and on being questioned, said it tasted like tobacco.

3. One of the gentlemen then bore his weight on the great toe of my right foot—protected only by a sock and woollen slipper—until I could no longer endure the pain. The countenance of Mr. S. remained quiet, and he did not complain on being questioned, but was observed to raise his right foot and turn his toes inward as he had done before.

*Experiments on the sensibility to caloric.*

I held my hand and arm for some time in contact with a mass of ice. He manifested, when questioned, no feeling of coldness, but showed slight muscular uneasiness and contortions of the mouth. At length when the pain of coldness had become very severe, he became quite composed, and said, on being questioned, that he felt well.

2. I held in my hand a smoothing iron, so hot that it raised a blister, and could not be borne by Mr. Greene, on whom it also rested. It produced on Mr. S. no particular effect; some contortions of the mouth only occurred.

*Experiments on colors.*

1. He being closely blindfolded by Mr. Foote, a broad lead colored surface, strongly illuminated, was placed before me, and I gazed intently upon it, when he, on being asked its color,. pronounced it "green." I then looked at a bright red, and he declared it, first, a "light color," then "white." To this succeeded a white surface, which he pronounced "yellow." Then a black, on which he said, "don't know"—"dark"—"something on it"—"dark."

I now nearly closed my eyes and turning them towards the ceiling of the room, endeavored, without involving myself in darkness, to exclude every vivid color, and also sought to have my mind occupied on the idea of color in the abstract, and not on any particular tint: his first answer was, "light"—then "sun"—then "pink." This experiment could be verified by the gentleman present, in this, that he answered "pink" when nothing of that color was in sight, but *they* could not know but what the idea of pink was in my mind; and therefore, although I have incorporated it into the report, I do not ask it to be put on a level with the other facts. In such an investigation as this, the declaration of no individual as to what was or was not passing in his mind, should be received as incontrovertible.

*Experiments on the sense of taste.*

These were sixteen in number, and so conducted, that no one present but myself knew, except in two or three instances, what I tasted, until the whole were completed. The different glasses used in the experiments were numbered, and the contents of each were registered in a sealed paper, which, after the tasting was over, was opened and found by the examination of Mr. Foster and Mr. Buchanan to be correct. I rinsed my mouth after each experiment, and sometimes used a brush—in short, did not proceed to a subsequent glass, till the taste produced by a preceding, when, indeed, there was any, had died away. Some of the glasses were empty.

1. Milk—"*Good*"—"*don't know.*"
2. Water—"*Don't like it*"—"*sour.*"
3. Empty—"*Well*"—"*doesn't taste like any thing*"—"*don't like it.*"
4. Wine—"*Don't taste*"—"*don't know.*"
5. Peppermint water—Muscular action of the face—"*Don't know.*"
6. Syrup of loaf sugar—"*Very good.*"
7. Water—"*Wine.*"
8. Vinegar—On being asked by Mr. B., "Is it sour?" answered, "*Yes.*"
9. Brandy—"*Don't like it*"—"*don't know*"—"*makes me sick.*"
10. Sweet cake without fruit—"*Likes it.*" On being asked what kind of fruit it was, answered—"*Resembles raisins.*"
11. Infusion of nut-gall—"*Good*"—"*don't like it.*"
12. Strong salt and water—"*Tastes nothing*"—"*water.*"

13. Piece of cold chicken—"*Don't like it.*"
14. Radish—"*Nothing.*"
15. Empty glass—Contortions of the mouth—"*Don't like it.*"
16. Strong, bitter infusion of Colomba root—"*Good*"—"*tastes sweet.*"

*Second Series of Experiments, Saturday night, May* 14.—Present Mr. Selves, to be mesmerised, and Mr. Burdsal, Mr. Foster, and Mr. Thos. J. Matthews, professed believers in the extraordinary powers and susceptibilities of one in a state of mesmeric sleep; also Messrs. Hall and Buchanan as reporters of the phenomena, and Dr. Warder, Mr. Symmes, and Mr. McGuffey, as spectators, with a few ladies.

*The Mesmerising.*

At 31 minutes past 8 o'clock, Mr. S. took his seat, with a pulse beating 80 in a minute. Under the direction of Mr. Burdsal, I undertook to throw Mr. S. into somnambulism, by pressing my thumbs upon his, and looking him steadily in the eyes. In 4 minutes he was asleep, with considerable rigidity of his limbs; his breathing natural. He was then blindfolded.

*First Experiment.*

My design was to observe his movements when there was perfect silence in the room, and he was left to himself untouched. I did not make any effort to act on him, but took a chair a few feet behind him, to watch. I then went into the adjoining hall, and afterwards out of the house. He slowly raised, first one hand, and then both; then made an effort to rise from his chair; spread out his arms; leaned forward, and had contortions of the face. This experiment lasted ten minutes.

*Second Experiment.*

I now caused Mr. Mansfield to take hold of his hand and press it in a variable manner; he also rubbed the hand, wrist and head. I remained in the room to witness the effect. He raised the left foot, and then the right, and kept both about ten inches from the floor. This experiment lasted 5 minutes.

*Third Experiment.*

I called audibly to Dr. Warder to be put in connexion with Mr. S.; he came forward, but I placed Judge Hall's hand, instead of the Doctor's, in Mr. S.'s, and made the prescribed passes, to connect them together. Dr. Warder then put *several* questions to him, which he answered. The Judge did not speak, and the Dr. did not touch him; at length Mr. Burdsal said, he is not answering Dr. Warder, after which we could get no further replies.

### *Fourth Experiment.*

This was on taste. I was myself the taster, no person save Mr. McGuffey and Mr. Foster knowing what I took. Three successive trials were made with each of the following articles, it being tasted anew twice. In the first trial, I put no leading question—in the second the questions led off towards some other article—in the third, they led towards the article, or in a different way. What seemed to be ample time for distinctness was allowed. The following were the answers, as recorded by Mr. Buchanan, and distinctly heard by all who were near him. The articles were numbered. The answers in the first column were obtained without leading questions; those of the second and third columns by leading questions—the former directed from, the latter to the article tasted.

| | | | |
|---|---|---|---|
| 1. Bread and butter. | Screws up his mouth and smacks his lips—'*Sweet.*' | '*Don't like that.*'—Same movements of the face. | '*Tastes like bread.*' Less movement of the face. |
| 2. Hydrant water. | Screws up his mouth —'*Water.*' | Twists his mouth—'*Weak.*' | But little movement of the face—'*Strong.*' |
| 3. Boiled ham, cold. | Face placid, smacks his lips—'*Good;*' '*don't know.*' | Twists his mouth—'*Don't know;*' '*don't like that.*' | Smacks his lips—'*Can't taste that.*' |
| 4. Hydrant water. | Contortions of the face & mouth—'*Don't like it.*' | Contortions of the mouth—'*Don't like it;*' '*tastes salt.*' | Smacks his lips—'*Don't know.*' |
| 5. Boiled peas, warm. | Face placid—'*Yes;*' '*good.*' | Smiles, then screws his mouth—'*Don't like that.*' | Contortions of the face—'*Don't like that.*' |
| 6. Hydrant water. | Face placid—'*Yes;*' '*very well.*' | No change of expression—'*Can't taste that.*' | Contortions of the face—'*Nothing.*' |
| 7. Boiled egg. | Hard compression of the lips—'*Don't like it;*' '*don't know.*' | Lips compressed—'*Don't like it;* ' '*tastes bitter.*' | No expression of face—'*Can't taste that.*' |
| 8. Hydrant water. | Looks pleased—'*Good;*' '*don't know.*' | Face placid—'*Water.*' | Same expression—'*Can't taste that;*' '*can't tell.*' |

*Fifth Experiment.*

This was on sight. Placing my back towards him, I looked intently on four successive articles, in a strong light.

The first was a common counter dust brush. Quest. Do you see what I am looking at? Ans. "*Something there.*" To other questions concerning it, not of a leading kind, his replies were—"*It opens*"—"*it pulls out there*"—"*something on there,*" and "*I don't know.*"

The second article was a red morocco razor case, with the cap off, and one of the razors partly drawn out. His replies to various questions were—"*It looks bright*"—"*on there*"—"*on that*"—"*on the table*"—"*I don't know.*"

The third article was a small plaster cast of a head, designed for phrenological purposes:—His answers to different questions were—"*Down there*"—"*round*"—"*don't know*"—"*hard*"—"*don't know.*"

The fourth article was a small, red carpet bag. Answers to various questions—"*Can't see that*"—"*don't know that*"—"*something on there.*" To the question, do you see me? ans. "*Yes.*"

*Sixth and last Experiment.*

Standing behind Mr. S., I took Mr. McGuffey on my shoulders. Quest. What do you see? "*Don't like that*"—"*it hurts.*" Where? "*Down there,*" and appears disturbed. Does it hurt your foot? "*Yes.*" The left foot? No answer. Becomes more quiet. Would you like to go home? "*Yes.*" Have you eat or drunk more than you wanted? "*No.*"

His pulse at this time was 80 in a minute and regular. On raising his eyelid, the pupil was dilated, but contracted under a strong light. Awakened after an hour and forty minutes.

It will, I think, be granted, that in these two series of experiments Mr. S. did not give evidence of sympathising with me in my bodily sensations. The few answers which seem to favor the conclusion that he did, may, I think, be otherwise explained. 1. If a man blindfolded, should have been called on to answer similar questions, according to the doctrine of chances *some* of them would be correct. Thus, when four colors were successively observed, and there were scarcely more than twice that number to draw answers from, it would have been very remarkable if all should have been erroneous, yet he named but one of the four, and did not decline speaking of the others, but declared them different from what they were; and when on the first night three kinds of food were tasted each once, and on the second, four kinds were each tasted three times, making fifteen in all, it might have been expected that much more than one would have been correct; but such was not the fact, and even that one was imperfect, for when I tasted bread and butter, he only said, "*tastes like bread,*" but immediately before had said of the same article, "*don't like it.*" Again, water was tasted, in the experiments of the two nights, fourteen times, and as it was announced to be a drink, we might expect that the answer would be correct once or twice. It was, indeed, correct twice; but immediately after one of these answers, he said, on another tasting, "*strong;*" and in other experiments with water from the same hydrant, he said, "*don't like it,*" "*tastes salt.*" In another experiment he said of it, "*don't like it*"—"*sour*"; in another, "*wine.*" Of milk and a syrup of loaf sugar he said "*good,*" "*very good,*" which would seem to indicate, that he was impressed by what acted on my organs of taste, but I am inclined to put these answers down to chance, when I recollect that he said of a very strong solution of common salt, "*taste nothing*"—"*water*"; of a boiled egg that it was "*bitter,*" and of an intensely bitter infusion of Colomba root, "*good*"—"*tastes sweet.*"

2. Some of his answers, as would be those of a person blindfolded, appeared to be brought out by the mode of interrogation. Thus when I had vinegar in my mouth, Mr. Burdsal, *by accident* (for he did not know), asked him if it was sour? and he answered "*yes,*" but still did not give the name. Again, when I was eating cake, and asked him what *fruit* it was like, he answered "*raisins.*" Again, when my eyes were partially closed, and nothing of a definite color acted on them, but I kept on asking what color I saw, he answered "*pink.*" Again, in the experiments of the second night, when I tasted the same articles three successive times, changing my *mode* of interrogation each time, his answers changed likewise, so that in no case were they uniform. Now I think these variations in his replies can only be ascribed to variations in the mode of questioning.

3. His apparent sufferings under some of the inflictions on me, may be explained on the principle of coincidence. As soon as he was put to sleep, the second night, and left to himself, no one touching him, and the room in perfect silence, he commenced and maintained a series of muscular movements of his face, arms, legs, and body. These then may be regarded as phenomena running through all the experiments, and sometimes coinciding with them in time, so as to seem a part of them. Thus when I had a heavy weight on my shoulders, and nothing was said to him about taste, the muscular movements which he exhibited might have occurred, if no such weight had been borne by me; and when a gentleman rested his weight on my great toe and stood silent, and he moved his foot, he might have done it, if I had not been acted on, seeing that such motions occurred spontaneously several times. These automatic muscular movements were, in fact, a kind of constant quantity, running through all the experiments, and by coincidence and contrariety, harmonising with some and proving discordant to others, and to be rejected from the results of the whole.

4. It is probable that some of his answers were modified by what he heard around him. That he did hear those who were not in connection with him the first night, seemed almost certain, from the fact that his contortions, writhings, and signs of pain, became more and more violent, the harder a gentleman rubbed his coat sleeve with sand paper, while my arm was rubbed with a piece of fur. But the second night it was *demonstrated* that he heard, for he answered several questions put by Dr. Warder, whom he believed to be in communication with him, when he was not.

Thus I have pointed out four circumstances, by which we may explain the few correct replies obtained from him; and, I repeat the conclusion already stated, that he did not feel the impressions made on my system. But I go *no* further; I do not say that he has not felt those made on the systems of other persons; I do not say, that other mesmerised persons do not feel all that is felt by those placed in communication with them. I do not say, because Mr. S., under my experiments, could hear those *not* in connection with him, that he can do it at other times, or that other persons in mesmeric somnolency can hear what is spoken in their presence. I disclaim all intention of drawing conclusions relative to him at other times, or others at any time.

